# Changes in sexual behavior among high-school students over a 40-year period

**DOI:** 10.1038/s41598-021-93410-6

**Published:** 2021-07-07

**Authors:** Catrin Borneskog, Elisabet Häggström-Nordin, Christina Stenhammar, Tanja Tydén, Stavros I. Iliadis

**Affiliations:** 1 Department of Nursing and Midwifery, Northumbria University, Newcastle Upon Tyne, NE7 7XA UK; 2 School of Education, Health and Social Studies, Dalarna University, Högskolegatan 2, 791 88 Falun, Sweden; 3 School of Health, Care and Social Welfare, Mälardalen University, 722 20, Västerås, Sweden; 4 Department of Women’s and Children’s Health, Uppsala University, 751 85 Uppsala, Sweden

**Keywords:** Epidemiology, Public health

## Abstract

The aim of this study was to investigate sexual behavior, contraceptive use, risk factors as well as sources of sex information among first-year high-school students in Sweden. Secondly, to assess differences between genders and study programs as well as changes over a 40-year period. A repeated cross-sectional survey was conducted in two cities. A questionnaire comprising 77 items was used. The study population consisted of 415 students (63.4% females). The median age of sexual intercourse was 15 years. In total, 37% had had sexual intercourse, compared to 56.3% in 2009 and 45% in 1999 (*p* < 0.001), and the proportion of students who had their first sexual intercourse was not influenced by gender. More students in vocational programs (46.3%), compared to theoretical (33.3%), had experience of at least one sexual intercourse (*p* = 0.019). The same extend of contraception use at first and latest intercourse was reported, compared to previous studies. Forty-nine percent were mostly informed about sex from the internet, while in previous years, magazines, family and youth clinics were the main information sources. Comparing over time, students were in general less sexually experienced and less engaged in non-penetrative sex and physical intimacy. These findings call for a new approach, when designing sex and relationship education and health-care counseling in adolescents.

## Introduction

Sweden has a wealth of research and knowledge on adolescent sexuality, sexual behavior and the use of contraception^1^. Public policies have focused on improving sexual health and a constant effort towards this direction is crucial, to keep up with societal changes^2^.

The median age of first sexual intercourse (FSI) has stayed relatively steady over the past thirty years, reported at around 15.5 years for about 50% of 16-year-olds in Sweden^1,3–8^. Similar data have been reported from the US, Greece, Ireland and Portugal^9–12^. Previous Swedish studies have shown that female 16-year-olds have more sexual experiences than their male peers do^1,4^. In Sweden, the use of contraception among young individuals has traditionally been high for the last thirty years, as more than two thirds of 16-year-olds reported using contraception at their first sexual intercourse^4,5,13^. However, in a recent Swedish study on adolescents’ sexual development and transition to adulthood, an inconsistent use of contraception was found^7^. Chlamydia trachomatis is the most commonly reported sexually transmitted infection (STI) among young people and it can lead to severe reproductive complications^14^. Sustained prevention of STIs and promotion of safe sex behavior remain the key priorities within the field of adolescent sexual and reproductive health and preconception care^1,15–17^. An over sixty year long tradition of sex and relationship education as well as easily accessible youth clinics, so called ‘youth friendly health-care services’^18^ have facilitated an open and supportive attitude to adolescents’ sexuality in Sweden^19^. In addition to sex education as a facilitator of sound and healthy attitudes to sexuality, it has been shown that people’s attitudes to sexuality and sexual behavior change concurrently with changes in society^1^. The introduction of the internet in the late 90’s and the development of social media since the millennium are illustrative examples of unceasing evolvement. The impact of social media has affected on people’s lives in different ways and the effect is perhaps greatest in today’s adolescents who were born during the 21-century and never have experienced life without internet access^20^.

The easy access to pornography via the internet is a source of worry as it may alter the user’s mental, emotional, and social traits^21–24^. Pornography consumption may contribute to creating a hostile environment at school, sexual harassment and sexual violence^25–27^, and has to be viewed as a potential threat to adolescent health and wellbeing. In fact, almost all teenage boys and more than 50% of teenage girls have consumed pornography^28–30^.

It is important to monitor trends in adolescent sexual behavior as those will affect the individual’s sexual and reproductive health and possibly psychological health and wellbeing later in life. Therefore, aspects such as first sexual intercourse, access to pornography and sexually transmitted infections should be taken into careful consideration when reviewing sex and relationship education and health-care counseling. In most countries, school is the main platform for sex education and an up-to-date approach to adolescents through prevention strategies and information on sex related aspects, based on current trends in sexual behavior, is essential^4^. In fact, the efficacy of information and prevention programs has been proven in periods of economic stagnation, when less extensive sex education programs and cutbacks in prevention inevitably led to negative trends regarding STIs and teenage abortion rates^4^. Ongoing research and continuous assessment and follow-up of information and prevention strategies are therefore essential^1^.

The aim of this study was to investigate sexual behavior, contraceptive use, risk factors as alcohol as well as sources of sex information, among first-year high-school students in Sweden. Secondly, to assess possible differences between genders and study programs as well as changes over a 40-year period.

## Results

The study population comprised 415 high school students, 255 females (63.4%) and 146 males (36.3%). Among study participants, 300 (72.3%) followed a theoretical program and the remaining vocational courses. Class attendance was high according to school teachers and the participation rate was 98% of students present in the class at the day of the survey. An online survey was returned by 91% of participants and 9% chose to complete a printed questionnaire. The internal dropout rate was 1%.

The median age of the participants was 16 years (min–max; 15–20) which did not differ between genders and study programs, and 5.1% of students were 18 years or older. In Uppsala, 89.3% of participants studied a theoretical program while in Västerås the corresponding figure was 50.5% (*p* < 0.001, χ^2^). The rate of study participants born abroad did not differ between the two cities, and almost one-third of all participants had at least one parent who was non-Swedish born, figures being in line with the national statistics^31^. In total, 22.5% reported occasional or regular tobacco use and nearly half of these smoked cigarettes (Table 1). Eighty-five participants (21.4%) reported being in a relationship at the moment of the survey. One-hundred three students (74.6%) considered themselves as mature enough at first sexual intercourse.

**Table 1:** Background characteristics of participating first-year high-school students.

	n (%)
City	
Uppsala	233 (56.1)
Västerås	182 (43.9)
Total	415
Gender	
Female	255 (63.4)
Male	146 (36.3)
Total	401
Program	
Theoretical	300 (72.3)
Vocational	115 (27.7)
Total	415
Participants’ country of birth	
Sweden	331 (84.9)
Other	59 (15.1)
Total	390
Parents’ country of birth	
Sweden	278 (67.3)
Other (at least one of parents)	135 (32.7)
Total	413
Feel like a person of same sex as my birth sex	
Partly/totally agree	378 (95)
Partly/totally disagree	20 (5)
Total	398
Tobacco products	
No	307 (77.5)
Yes	89 (22.5)
Total	396

In the total sample, 37% reported that they had had at least one sexual intercourse and 82.5% of those had sexual intercourse more than once, with the same or other partner. The proportion of students who had had their first sexual intercourse was not influenced by gender; however, more students in vocational programs had had at least one sexual intercourse compared to their fellows in theoretical courses. After further stratifying for gender, it was shown that this association was driven by girls in vocational programs, who had more often had their first sexual intercourse than girls did in theoretical courses. The median relationship duration before sexual debut was two months (min–max; 1 week–2 years) and no differences were observed between boys/girls or study programs, whereas the median age at first intercourse was 15 years for the study participant and 16 years for their partner, regardless of gender (Table 2).

**Table 2:** Sexual experience and related parameters among first-year high-school students in total, as well as stratified by gender and study program.

	n (%) or median (min–max)	p^a^
Sexual intercourse ever (total sample)	143 (37)	–
Sexual intercourse ever		ns
Males	53 (38.7)	
Females	89 (36)	
Sexual intercourse ever		0.019
Theoretical	93 (33.3)	
Vocational	50 (46.3)	
Sexual intercourse ever (males only)		ns
Theoretical	39 (36.4)	
Vocational	14 (46.7)	
Sexual intercourse ever (females only)		0.022
Theoretical	53 (31.2)	
Vocational	36 (46.8)	
Age at first intercourse	15 (9–19)	ns
Males	15 (9–19)	
Females	15 (10–17)	
Age of partner at first intercourse (partners of male students)	16 (13–32)	–
Age of partner at first intercourse (partners of female students)	16 (12–23)	–
Condom at first sexual intercourse	82 (58.6%)	–

^a^Pearson chi-square or Fisher’s exact test.

Overall, differences in sexual behavior and related risk factors between male and female students were observed only in a few variables; a higher proportion of girls reported history of STI and more girls than boys had talked with their parents about sex. Regarding sexual activities other than intercourse, the only significant difference was observed in the question “have you ever masturbated”, where more boys than girls answered positively (Table 3).

**Table 3:** Sexual behavior and risk factors across genders and study programs among first-year high-school students.

	Males	Females		Theoretical program	Vocational program	p^a^
	n (%) or median (min–max)		p^a^	n (%) or median (min–max)		
Median number of sexual partners^b^	2 (1–30)	1 (1–14)	ns	1 (1–19)	1 (1–30)	ns
Steady relationship	29 (20.3)	56 (22.1)	ns	52 (18.3)	33 (29.2)	0.021
Contraception at first intercourse^b^	44 (83)	66 (76.7)	ns	79 (86.8)	32 (65.3)	0.015
Ever had sexually transmitted infection (suspicion)^b^	8 (15.7)	21 (24.7)	0.008	16 (17.8)	13 (27.7)	ns
Alcohol use at first intercourse^b^	10 (18.9)	15 (17.6)	ns	19 (20.9)	4 (12.5)	ns
Tobacco use (regular or occasional)	40 (28)	49 (19.5)	ns	64 (22.5)	25 (22.3)	ns
Parents have talked about sex	62 (45.6)	146 (60.1)	0.007	141 (52)	67 (60.9)	ns
HIV/AIDS fear influenced own attitude about sex	20 (14.2)	41 (16.3)	ns	43 (15.2)	18 (16.2)	ns
HIV/AIDS fear influenced others’ attitude about sex	41 (28.5)	83 (32.8)	ns	78 (27.4)	46 (40.4)	0.032
Ever masturbated	123 (88.5)	169 (71.6)	< 0.001	215 (78.5)	79 (76)	ns
Ever kissed	102 (73.9)	167 (67.9)	ns	196 (70.5)	75 (68.8)	ns
Petting	37 (27.6)	80 (33.2)	ns	80 (29.3)	38 (36.5)	ns
Oral sex; given	38 (28.1)	75 (31.1)	ns	78 (28.5)	36 (34.6)	ns
Oral sex; received	39 (28.7)	70 (29.2)	ns	71 (25.8)	39 (37.9)	0.03
Anal intercourse ever	8 (6.3)	17 (7.1)	ns	14 (5.3)	11 (10.7)	ns

^a^Pearson chi-square, Fisher’s exact test or Mann–Whitney.

^b^Calculated among those who have had intercourse.

Regarding pornography, 275 (70.3%) of students reported having watched porn at least once and, of those, 155 (59.4%) did so regularly. In both questions, males were overrepresented compared to females (males vs. females, 88% vs 60.3% ever watched porn; 86.5% vs. 34.8% regular use; both *p*-values < 0.001, χ^2^) and no differences were observed between study programs.

Results from previous similar studies^4–6,8^ in Swedish high-school settings were compared to the present study (Table 4). A lower proportion of students in our study had at least one sexual intercourse, compared to students from the last three studies. Likewise, participants in the present study were less sexually experienced and engaged in non-penetrative sex and physical intimacy than students in 2009. On the other hand, the same extend of contraception use at first and latest intercourse was reported in 1999, 2009 and 2019 and was higher than earlier years. Fewer students in 2019 were influenced in their own attitudes about sex by HIV/AIDS compared to previous years.

**Table 4:** Differences in sexual behavior and risk factors among first-year high school students over time.

	1979 (n = 181)	1989 (n = 383)	1999 (n = 408)	2009 (n = 384)	2019**–**2020 (n = 401)	p^a^
	N (%) or median (min–max)					
Sexual intercourse ever	71 (39)	179 (47)	189 (45)	209/371 (56.3)	143/387 (37)	< 0.001
Median number of sexual partners		2 (NA)	2 (NA)	2 (NA)	1 (1–30)	
Steady relationship	139 (77)	104 (27)	97 (24)	97/382 (25.4)	85/397 (21.4)	< 0.001
Contraception at first intercourse^b^	45 (65)	113 (63)	144 (76)	170/209 (81.3)	111/140 (79.3)	0.02
Contraception at latest intercourse^b^	55 (77)	121 (68)	155 (82)	176/209 (84.2)	104/128 (81.3)	< 0.001
Ever had sexually transmitted infection^b,c^		7 (2)	13 (7)	42/206 (20.4)	29/137 (21.2)	< 0.001
Alcohol use at first intercourse^b^	36 (52)	52 (30)	44 (23)	36/208 (17.3)	25/139 (18)	< 0.001
Regular cigarette use	–	77 (21)	57 (14)	50/384 (13)	15/381 (3.9)	< 0.001
Parents have talked about sex	–	221 (59)	213 (51)	207/381 (54.3)	208/381 (54.6)	ns
HIV/AIDS fear influenced own attitude about sex	–	206 (54)	164 (40)	92/383 (24)	61/394 (15.5)	< 0.001
HIV/AIDS fear influenced others’ attitude about sex	–	–	230 (56)	115/383 (30)	124/399 (31.1)	< 0.001
Ever masturbated	–	–	–	296/366 (80.9)	294/378 (77.8)	ns
Ever kissed	–	–	–	308/373 (82.6)	271/387 (70)	< 0.001
Petting	–	–	–	285/375 (76)	118/377 (31.3)	< 0.001
Oral sex; given	–	–	–	157/369 (42.5)	114/378 (30.2)	< 0.001
Oral sex; received	–	–	–	165/370 (44.6)	110/378 (29.1)	< 0.001
Anal intercourse ever	–	–	–	49/370 (13.2)	25/369 (6.8)	0.004

^a^Pearson chi-square, Fisher’s exact test.

^b^Calculated among those who have had intercourse, NA = not available data.

^c^In 1989 and 1999 the wording was: ‘Did you ever have a STI diagnosed?’ while in 2009 as well as the present study the question was ‘Have you ever suspected that you had a sexually transmitted infection?’.

**Figure 1:**
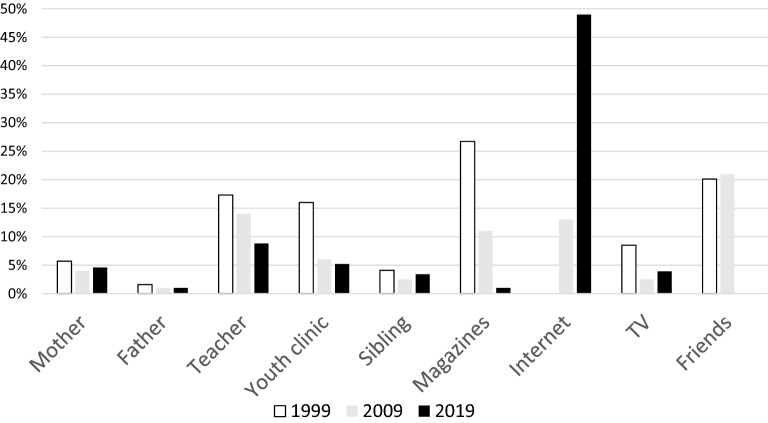
Primary source of information about sex among first-year high schools students in 1999, 2009 and 2019.

Figure 1 illustrates the primary source of information about sex among high school students in 1999, 2009 and 2019 and comprises the present study’s results as well as data from previous studies^4,5^. Almost half of the participants in the present study were mostly informed about sex from the internet while in previous years, magazines, family and youth clinics were the main sources of knowledge on sex issues. However, family still appears to contribute with information about sex, at least to some extent, since 54.6% of students in our study reported that they had discussed sex with their parents. Moreover, over half of the students (56.8%) felt that they had received adequate information about sex at school, with a higher proportion of boys sharing that opinion. Notably, the corresponding figure in the 2009 study was 70%^4^.

## Discussion

The aim of this study was to investigate sexual behavior, contraceptive use, risk factors and sources of sex information, among first-year high-school students in Sweden. Furthermore, differences between genders and study programs as well as changes over a 40-year period were assessed. The most interesting finding in our study was the clear decline in the number of students having had their first sexual intercourse at the age of 16 years, compared to previous studies in similar settings^4–6,8^. The same trend have also been reported from the US and Asian countries^9,32^. In the present study, we observed a declining trend in adolescent alcohol use (Table 4), which may, at least partly, explain this finding^1^. Another possible explanation is that many adolescents engage in internet and social media interactions. These contacts and relationships might theoretically delay the need for physical contacts for 16-year olds. Current research indicates that online sex education sources may influence youth sexual behavior, in terms of a safer practice^33^. Divecha et al. reported how youth learning about sex through social media resulted in increased self-efficacy to use condoms, as well as greater knowledge of STI and HIV risk factors^34^. Our results are thus interesting and might point to a new trend regarding the level of sexual experience among adolescents; however, further studies on a national scale are required to elucidate this issue. Nevertheless, it is reasonable to assume that the internet and social media are affecting people’s social and intimate behavior and interactions.

The median age at FSI was 15 years in our population. Results from a large Scandinavian survey, including over 30 000 Swedish women, showed a median age of 16 at FSI in women born 1989–1994, thus depicting trends in a population slightly older and with a wider age span compared to our study’s^35^. Similarly, the mean age of first sexual intercourse was 16.4 (± 1.8) years in a large study of Portuguese students^36^. According to a report from the Public Health Agency of Sweden, based on data from 2009–2014, the average age at FSI in Sweden was 16.1 for girls and 16.6 for boys^1^. It should be kept in mind that our study focused on a specific population of individuals with a small age range, compared to the studies named above.

The finding of a higher proportion of students with sexual debut in vocational programs has been demonstrated in a previous study^4^. In the present results, this finding was most prominent among girls; however, a similar tendency was also seen among boys but it did not reach statistical significance, possibly due to lack of power. Moreover, more students in vocational programs reported living in a steady relationship, compared to those in theoretical. The choice of a vocational program is possibly associated with the students’ socio-economic background. In an Irish study, socio-demographic and lifestyle factors were stronger predictors of age at FSI among girls than boys^37^. A pursue of family initiation at a younger age, instead of university and career plans, might explain this observation; this is further supported by the higher contraception use prevalence at FSI in theoretical programs^3–5,38^. Indeed, the use of contraception at first and subsequent intercourse was steadily high in our study. A large Scandinavian study assessing over 11 000 women reported a contraceptive non-use at FSI in 16.6% of Swedish women, a result comparable to our findings^39^. On the other hand, a systematic review and meta-analysis of contraceptive use among unmarried females reported a 57.6% rate of contraceptive use at FSI worldwide, a figure lower than our results^40^. Similarly to our study, condom was the predominant contraceptive method. One may conclude that sex and relationship education in Sweden has been successful and an increased awareness on the importance of good sexual and preconception health is evident among adolescents.

More girls than boys had a history of STI. However, analyses were made in few individuals. Moreover, this finding needs to be viewed in the light of the fact that more girls than boys are visiting youth clinics where they are being offered STI testing as a routine, and consequently more girls are diagnosed with an STI^41^. Students in this study did not appear to be worried about HIV/AIDS transmission. This is a clear change in attitudes compared to our previous studies. Possibly, this is a result of the low incidence of people with HIV today, combined with the knowledge of the effectiveness of antiviral drugs^42^.

Another substantial change from previous years was the information sources used by students on sex and sexuality. Perhaps a natural development in the 21-century, using the internet as the main information source was paramount, in relation to any other source. The low number of 16-year-olds preferring a youth clinic for this purpose should be considered, when planning future sex and relationship education and sexual and reproductive health strategies. In Sweden, an internet based youth clinic was introduced in 2008^43^. This service is called ‘the youth clinic on the net’ or ‘Youmo’ and has been translated into several languages. The clinic is run by the Swedish Association of Local Authorities and Regions (SALAR) and provides evidence-based, non-profitable information and sex and relationship advice to adolescents. Perhaps technology-based interactive applications may be required to modernize the supply of sex and relationship information to young people^43^. In fact, it might be so that visiting a clinic is becoming an outdated practice as a mean of retrieving sexuality-related information, and will only remain as a center that addresses health problems.

On the other hand, despite the obvious advantages of internet, the lack of tight protective filters for inappropriate sexual content and insecure information sources is worrying. Furthermore, pornography is largely gender-stereotyped, depicting both male and female sexual activity in a biased and victimized way, and saying little about emotions and sexual relationships in individuals. In our study, nearly three quarter of students had watched pornography at least once. Stanley et al. found that negative gender attitudes among boys overlap with regular use of online pornography and it has been argued that pornography is both underpinned by and perpetuates gender inequality^25^. Young people are informed and active consumers of a wide range of media, and sex education should draw on that expertise to encourage critique and gendered understandings of pornography and its issues^25^.

The two-step cluster selection of the study population should be considered as a study strength. The validity of the study answers was supported by the use of an updated survey previously utilized in several studies. Furthermore, students were unaware of the detailed study content prior to survey distribution, thus reducing the risk of biased answers and planned absence. Anonymity was secured and an online option of the survey was offered and chosen by the vast majority of students, which should have strengthened the validity of the answers. Furthermore, the printed questionnaires were revised manually and no overrepresentation of students in terms of age, gender, or country of birth was observed. Unfortunately, due to the low percentage of printed surveys, no statistical comparisons were possible. We have no reason to believe that responders would differ depending on the type of survey collected. Moreover, a test–retest pilot study with good reliability was performed^44^. This study was the largest in the study-series, providing enough power to detect differences between subgroups and allowing for comparison with previous surveys and a better understanding of modern trends in sexual behavior among adolescents. Data collection took place on regular school days. Moreover, nearly all enrolled students returned completed questionnaires.

However, this study has also some limitations. The inclusion process was partly obstructed due to some schools rejecting participation. Another unexpected complication was the COVID19 outbreak in March 2020, during the ongoing data collection, delaying the process until June 2020. However, since Sweden has followed an open-school policy during the pandemic, no schools were excluded from the study due to the pandemic.

Nevertheless, this made it necessary for the sampling to be purposive, aiming to match proportions in study program type and gender. Still, the study sample should be considered as representative, given the fact that the proportion of students in theoretical/vocational programs is in line with that of the local and national background population. Moreover, the proportion of study participants from the two cities was balanced, given the city population size. Another limitation is the over-representation of girls in the study sample, since almost two-thirds of study participants were females. This can be partially explained by the national over-representation of girls in theoretical programs and is a result of attempting to have a population-representative proportion of study programs. On the other hand, the main study variables were examined separately for boys and girls and in some cases, a gender stratification for specific variables was utilized, to account for this issue. However, the generalizability of our results should be interpreted with caution, as the original sample recruitment strategy could not be fully completed, due to the limitations named above.

## Methods

### Study design, settings and participants

This was a descriptive cross-sectional survey study, undertaken in first-year high-school classroom settings, where students from theoretical (study preparing) and vocational (vocational-technical) study programs participated. The study was carried out in two Swedish cities, Uppsala and Västerås, during winter/spring 2019/2020. Uppsala is a university city with about 172,000 inhabitants and Västerås a postindustrial city with approximately 127,000 inhabitants.

### Procedure and recruitment

In 2019, the total number of first-year high school students was 3147 in Uppsala and 2544 in Västerås. The proportion of students following theoretical programs was 77% in Uppsala and 66.2% in Västerås for the background population.

An inclusion procedure similar to previous studies by our research group^4–6,8^ was followed. In order to recruit a population-representative study sample, theoretical and vocational educational programs from high schools in both cities were included in the selection process, in a two-step cluster manner and in a proper proportion, to account for the fact that the average percentage of the background population following theoretical programs was approximately 70%. Six schools were initially allotted in each city, in order to include a sufficient number of students that would provide enough study power, taking into consideration potential dropouts as well as the sample size from the 2009 study (n = 384)^4^. In this initial recruitment round, some schools chose not to participate and the COVID19 outbreak in the spring of 2020 further complicated the recruitment strategy. Therefore, a second purposive selection of schools and classes was undertaken. After school inclusion, classes were randomly selected, until the selection exceeded the planned inclusion sample size of 500 students. In total, 14 classes (11 theoretical and 3 vocational) were included in the study.

An explanatory letter about the study was emailed to the head of the upper secondary schools in each city, asking for permission to carry out the study. After permission, school principals at the selected schools received information about the study and were asked for participation. Students were not informed about the data collection in advance; however, they received oral and written information about the study on the morning of the data collection and that participation and data collection was voluntary and anonymous. The session started with oral/written information about the ongoing and previous studies. The students were offered the option to complete the questionnaire online or via printed surveys. The online questionnaires were collected and managed using REDCap electronic data capture tools hosted at Uppsala University^45^. An automatically generated link, provided by the REDCap platform, was distributed to the participants upon inclusion.

The Swedish Ethical Review Authority gave ethical consent to the study (Dnr 2019/04,231). All experiments were performed in accordance with relevant guidelines and regulations. Informed consent was obtained from all participants.

### Questionnaire

A modified version used in previous studies^4^ was utilized, along with new areas, to mirror changes in society and the current context and reality of adolescents in Sweden. For the purpose of the present study, 77 questions were analyzed and covered the following areas; background characteristics (10 questions), relationship status (8 questions), knowledge and information sources on sex issues (6 questions), pornography consumption and attitudes (8 questions), sexual behavior and experience (32 questions), history of pregnancy or sexually transmitted diseases (5 questions), attitudes regarding HIV/AIDS (3 questions), as well as contraception use (5 questions). The possible answers were provided in multiple choice, yes/no, Likert-scale or open-ended format.

The survey was pilot-tested and re-tested in a small group of students (n = 14), similar to the proposed study population, with two weeks interval. A high degree of correlation between survey answers was found for all questions (Spearman’s correlation coefficient r_s_ = 0.8–1). Thus, no alterations were made.

### Statistics

The Statistical Package for the Social Sciences (IBM SPSS Statistics) version 27 was used and statistical significance was set at a *p*-value of < 0.05. Differences between males and females, educational programs and changes through time in sexual experience, sexual behavior and related factors were examined by use of Pearson’s chi-square or Fisher’s exact test for binary variables and Mann–Whitney test for non-parametrical data.

### Comparisons with studies from 1979, 1989, 1999 and 2009

The first in these repeated cross-sectional surveys was undertaken by Weiner et al. in 1979, including 181 first year high school students in Uppsala^8^. In this study, 48.6% were boys and 51.4% girls. In 1989, Klanger et al. followed-up the 1979 study, with a random sample of 383 students in Uppsala^6^. In this study, 64% of the students studied at theoretical and 36% at vocational programs, whereas 53% were boys and 47% girls. The third study was undertaken in 1999 and it comprised students from two cities, Uppsala and Västerås. In total, 408 students participated; 39% were boys and 61% girls. Regarding study programs, 59.1% attended a theoretical and 40.9% a vocational program^5^. Lastly, the study from 2009 included classes from two cities, Västerås and Örebro. In this study, 384 students (44.5% boys and 55.5% girls) were included and 59.6% followed a theoretical study program^4^.

## Conclusion

In this study, we found that fewer first-year high-school boys and girls had experienced their first sexual intercourse, compared to previous repeated cross-sectional surveys. Moreover, fewer individuals were sexually experienced and engaged in non-penetrative sex and physical intimacy. A shift was also observed in information sources about sex since every other student uses internet as the primary source of sex information, while in previous years, magazines, family, and youth clinics were the main information sources. These findings call for a new approach, when designing sex and relationship education and health-care counseling in adolescents.

## Data Availability

The data that support the findings of this study are available upon reasonable request from the corresponding author. The data are not publicly available due to privacy and ethical restrictions.
